# Reclaiming wellness: Key factors in restoring optimal well-being in the Canadian Longitudinal Study on Aging

**DOI:** 10.1371/journal.pone.0329800

**Published:** 2025-09-24

**Authors:** Mabel Ho, Esme Fuller-Thomson

**Affiliations:** 1 Factor-Inwentash Faculty of Social Work, University of Toronto, Toronto, ON M5S 1V4, Canada; 2 Institute for Life Course & Aging, University of Toronto, Toronto, ON M5S 1V4, Canada; Landmark University, NIGERIA

## Abstract

**Background:**

This study examines characteristics of older adults who have regained optimal well-being at the end of the three-year study. The definition of optimal well-being used in this study refers to having adequate social support, high levels of older adults’ subjective perception of their aging process, physical health, mental health, happiness and life satisfaction and being free of limitations in Activities of Daily Living (ADLs) and Instrumental Activities of Daily Living (IADLs), disabling pain or discomfort, severe mental illness or cognitive decline in the preceding year.

**Methods:**

A secondary data analysis was conducted using the first two waves of data from the comprehensive cohort of the Canadian Longitudinal Study on Aging (CLSA), a large, national, longitudinal study on aging. The sample included 8332 older adults who were not in optimal well-being at baseline and aged 60+ at time 2. Bivariate and multivariable binary logistic regression analyses were used to examine which baseline characteristics were associated with achieving optimal well-being approximately three years later.

**Results:**

The prevalence of optimal well-being at time 2 was higher among respondents who, at baseline, were younger, married, physically active, not obese, non-smokers, had higher income, without sleeping problems, diabetes, arthritis, osteoporosis, and achieved at least two of the four wellness domains (i.e., physical, psychological and emotional, social, and self-rated wellness) were more likely to be in optimal well-being at time 2 than their counterparts.

**Conclusions:**

Old age does not necessarily result in poor physical health, nor is a decline in well-being inevitable. Almost one in four respondents who were in less than optimal well-being at baseline regained well-being over the ensuing approximately 3 years. Further research could investigate the association between policies and programs and their support for older adults in regaining optimal well-being in later life after a period of suboptimal well-being.

## Introduction

Old age is frequently perceived through a negative lens. Aging is often associated with health decline or deterioration [1]. It has long been regarded as a disease [2] or a problem. [3]. Aging is frequently cited as the primary risk factor for poor health [4] including cardiovascular, neurodegenerative, and metabolic diseases [5,6]. Older adults often experience ageist stereotypes, causing excessive fear of aging or growing old, also known as gerascophobia [7,8]. Many people worry about changes in physical appearance, declines in physical and mental health and functioning, and social losses. A recent study has shown that people who believe they look younger than their age report more positive age-related experiences, such as being asked for advice, while those who perceive themselves as looking older report more negative interactions with others [9]. These ageist experiences have been shown to affect people’s health negatively, resulting in increased risk for dependency, illnesses, and reduced recovery from diseases.

However, the evidence suggests that older Canadians live longer and in better health than previous generations [10]. There is a burgeoning interest in understanding the factors associated with lifelong flourishing. Old age does not necessarily imply poor health. A robust body of literature suggests that older adults are more likely to enjoy good health and longevity by eating well, refraining from smoking and excessive drinking, exercising regularly, staying socially connected, preventing or managing chronic diseases, and taking steps to minimize the risk of falls and accidents [11,12]. Our previous longitudinal study also identified many modifiable lifestyle factors preserving optimal well-being in later life. These factors included remaining physically active, maintaining a healthy body weight, avoiding sleeping problems, not smoking, and engaging in social activities [13–15]. However, there is relatively few studies examining factors associated with regaining optimal well-being in later life after a period of struggling with health or mental health challenges [16]. The current study focuses on factors related to regaining optimal well-being among older adults. It may shed light on potential strategies to promote resilience and help older adults achieve optimal well-being in later life. If health and social systems can be better aligned with the needs of older adults [17,18], and focus on their strengths rather than deficits [19,20], older adults can thrive as they age and truly experience the best possible later life.

### Conceptual framework

**Fig 1:**
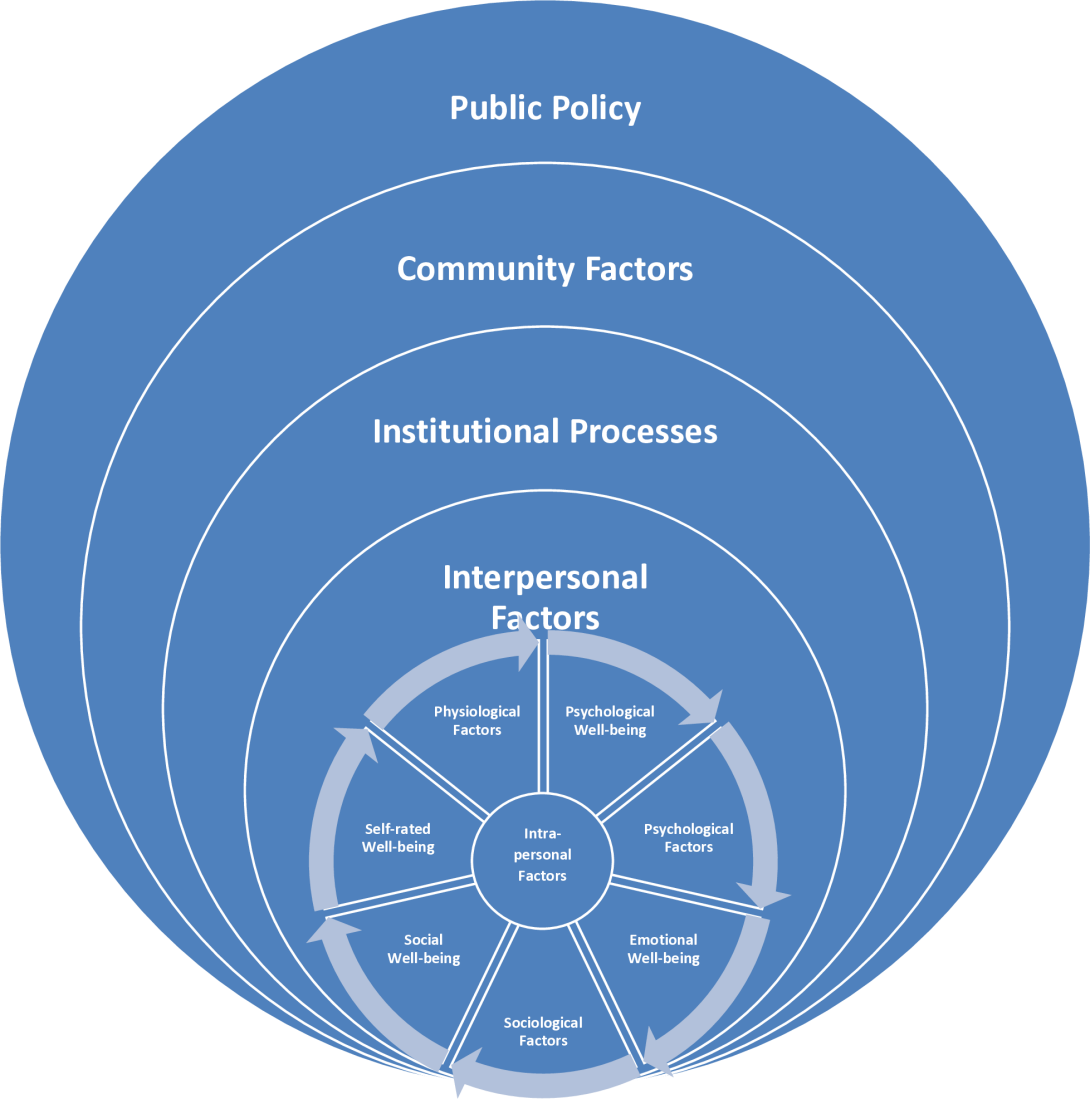
A conceptual framework used to study physical, psychological and emotional, social and self-rated wellness domains of optimal well-being.

The notion of optimal well-being used in this study is guided by a conceptual framework informed by three theoretical perspectives: ecological systems theory, [21,22] the concept of complete mental health, [23] and a multidimensional model of successful aging (Fig 1) [24]. The ecological systems theory [21,22] focuses on the relationship between people and their environments [25]. It highlights the importance of understanding how structural factors (e.g., economic, organizational, political and social environments) may affect the experience of aging. Two other frameworks provide helpful insights: Keyes’ concept of complete mental health [23], which views it as more than the absence of mental illness, but occurs at the intersection of emotional, psychological, and social well-being. It provides operational definitions of these three important elements. Young *et al.*’s multidimensional model of successful aging [24] suggests that successful aging is possible in the context of physiological limitations if compensatory psychological and/or sociological factors are considered. It suggests multiple pathways to foster optimal aging despite physical limitations. Combining these theoretical frameworks, the integrated definition of optimal well-being includes a combination of physical, psychological and emotional, social, and self-rated wellness, regardless of chronic conditions and physical disabilities. A more detailed description of the conceptual framework and definition can be found in our previous publications [13–15]. Therefore, optimal well-being is defined as a state wherein an older adult achieves a sense of physical, psychological and emotional, social, and self-rated well-being, regardless of chronic health conditions.

This definition of optimal well-being is based on both objective and subjective measures of aging well. Building on modified researcher-derived definitions of optimal well-being, the expanded definition of “successful aging”, hereafter referred to as optimal well-being, described in previous publications included not having limitations in performing activities of daily living (ADLs) and instrumental activities of daily living (IADLs), freedom from any severe mental illness, memory problems, and disabling chronic pain or discomfort, presence of adequate social support, and having high levels of the self-rated aging process, physical health, mental health, happiness and life satisfaction, regardless of the number of chronic diseases present [13–15].

### Study aims

Following a series of studies using the first two waves of the comprehensive cohort of the Canadian Longitudinal Study on Aging (CLSA) to examine how baseline factors (i.e., immigrant status, marital trajectories, and social participation) are associated with maintaining optimal well-being in a sample of those thriving at baseline [13–15], this study examines factors associated with regaining optimal well-being in later life by focusing on those who were not in optimal well-being at baseline and regained optimal well-being at time 2.

The study aims to address the following research questions:

1. Which domains of adversity at baseline (e.g., physical, psychological and emotional, social, and self-rated wellness) are most conducive to regaining optimal well-being?
2. What factors/behaviors/characteristics at baseline are associated with regaining optimal well-being approximately three years after baseline?

## Methods

### Study population

This study analyzed data collected at the time of recruitment (gathered between 2011 and 2015, hereafter referred to as baseline data) and follow-up 1 (gathered between 2015 and 2018, hereafter referred to as time 2 data) from the CLSA Comprehensive Cohort [26]. The Comprehensive Cohort was comprised of 30,097 Canadian men and women aged 45–85 years at baseline. Respondents were randomly selected through random-digit dialling [27]. To be eligible, they had to live within 25–50 km of 11 data collection sites across seven provinces and had to be willing to participate in the survey every three years for at least 20 years or until death. They were interviewed at home and underwent in-depth assessments at the data collection sites. Thirteen research ethics boards across Canada had approved the study protocol of CLSA. Details about the study are available at www.clsa.elcv.ca (accessed on 31 August 2024). CLSA was responsible for obtaining written consent from the respondents. The authors of this paper did not have access to the respondents’ identifiable information. This study, which involved secondary data analysis of CLSA data, was approved by the Health Sciences Research Ethics Board of the University of Toronto (protocol number: 38284, date of first approval: 18 October 2019, and date of recent approval: 7 October 2024).

The sample for the current study was restricted to those 60 years or older at time 2 who did not meet the criteria of optimal well-being at baseline. Among the 30,097 respondents at baseline [28], 27,799 respondents participated in time 2. Of these, 18,978 respondents were 60 years and over. Among these individuals, 10,375 respondents did not meet the criteria of optimal well-being at baseline. However, 2043 of these respondents had missing data on one or more variables included in the analysis. No individual variable had more than 5% missing data. The final sample consisted of 8332 respondents.

### Measures

#### Dependent variables assessed at both baseline and time 2.

***Optimal well-being*** Building on our previous publications, [13–15] respondents were classified as being in optimal well-being if they met each of the following four sets of criteria of (1) physical wellness (i.e., not having limitations in performing ADLs and IADLs, disabling chronic pain or discomfort); (2) psychological and emotional wellness (i.e., not having anxiety, depression, [29–30] post-traumatic stress disorder, [31] memory problems; never, rarely or some of the time feeling depressed; occasionally or all of the time feeling happy; and occasionally or all of the time feeling satisfied with life [32]); (3) social wellness (i.e., having someone to give advice about a crisis, having someone to show them love and affection, and having someone to confide in or talk to about themselves or their problems [33]); and (4) self-rated wellness (i.e., having self-rated aging process, physical health and mental health as good to excellent). Optimal well-being status was determined at both baseline and time 2. The sample for this study was restricted to individuals who did not meet the criteria for optimal well-being at baseline.

***Wellness Status Improvement from Baseline to Time 2*.** Improvement in four wellness domains was examined: (1) physical wellness improvement, (2) psychological and emotional wellness improvement, (3) social wellness improvement, and (4) self-rated wellness improvement. Wellness status was determined as described in the optimal well-being section at both baseline and time 2. Respondents who did not meet the criteria of a particular wellness domain at baseline but met the criteria at time 2 were classified as improving in that wellness domain. For example, respondents who did not have physical wellness (i.e., limitations in performing ADLs and IADLs, disabling chronic pain or discomfort) at baseline but achieved physical wellness at time 2 would be considered to have experienced physical wellness improvement.

#### Independent variable assessed at the baseline wave of data collection.

***Wellness Status at Baseline***. Wellness status in (1) physical, (2) psychological and emotional, (3) social, and (4) self-rated domains was determined at baseline.

#### Covariates.

Guided by the conceptual framework discussed in the review of literature section and based on a priori selection criteria within the constraints of variables available in the CLSA, a wide range of sociodemographic characteristics, chronic health conditions and lifestyle factors, all measured at baseline, were used in the analyses. These measures’ descriptions and the exact questions are available in S1 and S2 Tables in the Supporting Information [13].

### Statistical analyses

This study used SPSS Version 29 to conduct all analyses. The sample sizes presented are unweighted, while the percentages and odds ratios are weighted to address the probability of inclusion in the sample. The weights used for all analyses were created by the CLSA committee to be representative of the community dwelling population of middle age and older Canadians, taking into account unequal sampling probabilities, frame coverage error and non-response, to be in keeping with Statistics Canada weights [34]. The weighting variable was then rescaled to have a mean of 1 within this sample, a standard normalization technique used to prevent artificial narrowing of the confidence interval. Sample sizes are presented in their unweighted form. Bivariate analyses included chi-square tests and t-tests, which were conducted to compare baseline factors associated with those who achieved optimal well-being by time 2 versus those who remained in less-than-optimal well-being across the approximately three-year study period. A series of binary logistic regression analyses were conducted, with various baseline factors as predictors and changes in optimal well-being status from baseline to time 2 as the outcome. In Model 1, only wellness domains were included. In Model 2, age and sex were added to the model. In Model 3, all baseline covariates were added to the model. In addition, four wellness domain-specific analyses were conducted for respondents who did not achieve wellness of the specific domain at baseline, with various baseline factors as predictors and wellness status improvement in that domain from baseline to time 2 as the outcome. In the Full Model, all baseline covariates were added to the model. A significance level of 0.05 (**p* *< 0.05) was considered statistically significant for all the tests, and 95% confidence intervals (95% CIs) were provided in the logistic regression. The Hosmer-Lemeshow test was used to assess the goodness of fit, and the variance inflation factor was used to check for multicollinearity.

## Results

### Descriptive statistics

The characteristics of the final sample (*n* = 8332, unweighted counts) and chi-square statistics (weighted percentage) are shown in Table 1. Although all respondents in this analysis were not in optimal well-being at baseline, 23.4% met the criteria at time 2.

**Table 1:** Description of sample characteristics at baseline by optimal well-being status at time 2. Sample restricted to respondents not in optimal well-being at baseline (*n* = 8332).

Variables	Total		% who achieved optimal well-being	χ2 (df) p-value	Characteristics of those not in optimal well-being at baseline who regained optimal well-being at time 2 n = 1951	Characteristics of those not in optimal well-being at baseline who remained not in optimal well-being at time 2 n = 6381
	n = 8332	%				
**Physical wellness**						
No physical wellness	1788	21.5%	18.4%	31.9 (1), *p* < .001	16.9%	22.9%
Had physical wellness	6544	78.5%	24.8%		83.1%	77.1%
**ADL/IADL Limitation**						
No ADL/IADL limitations	7992	95.9%	24.0%	38.8 (1), *p* < .001	98.4%	95.2%
No ADL limitations, but IADL limitations	256	3.1%	9.4%		1.2%	3.6%
ADL limitations	84	1.0%	9.5%		0.4%	1.2%
**Disabling Pain**						
No	6770	81.3%	24.4%	19.6 (1), *p* <.001	84.7%	80.2%
Yes	1562	18.7%	19.1%		15.3%	19.8%
**Psychological and Emotional Wellness**						
No psychological and emotional wellness	4775	57.3%	16.1%	331.5 (1), *p* <.001	39.5%	62.8%
Had psychological and emotional wellness	3557	42.7%	33.2%		60.5%	37.2%
**Depression** (Having depression as classified by the CES-D score [29^–^30])						
No	6145	73.8%	27.1%	175.2 (1), *p* <.001	85.3%	70.2%
Yes	2187	26.2%	13.1%		14.7%	29.8%
**Felt Depressed**						
No	7303	87.7%	24.9%	68.1 (1), *p* <.001	93.0%	86.0%
Yes	1029	12.3%	13.2%		7.0%	14.0%
**Felt Happy**						
No	1499	18.0%	14.8%	75.5 (1), *p* <.001	11.4%	20.0%
Yes	6833	82.0%	25.3%		88.6%	80.0%
**Felt Satisfied**						
No	1923	23.1%	15.1%	95.6 (1), *p* <.001	14.9%	25.6%
Yes	6409	76.9%	25.9%		85.1%	74.4%
**Social Wellness**						
No social wellness	4549	54.6%	21.2%	27.6 (1), *p* <.001	49.4%	56.2%
Had social Wellness	3783	45.4%	26.1%		50.6%	43.8%
**Someone to give advice about a crisis**						
No	2878	34.5%	19.6%	36.4 (1), *p* <.001	28.9%	36.3%
Yes	5454	65.5%	25.4%		71.1%	63.7%
**Someone to show love and affection**						
No	1395	16.7%	11.3%	138.2 (1), *p* <.001	8.0%	19.4%
Yes	6937	83.3%	25.9%		92.0%	80.6%
**Someone to confide or talk to about a problem**						
No	2913	35.0%	18.1%	71.7 (1), *p* <.001	27.0%	37.4%
Yes	5419	65.0%	26.3%		73.0%	62.6%
**Self-rated Wellness**						
No self-rated wellness	1867	22.4%	13.9%	120.8 (1), *p* <.001	13.3%	25.2%
Had self-rated wellness	6465	77.6%	26.2%		86.7%	74.8%
**Self-rated Own Aging**						
Poor to fair	1054	12.7%	12.5%	79.8 (1), *p* <.001	6.8%	14.4%
Good to excellent	7278	87.3%	25.0%		93.2%	85.6%
**Self-rated Physical Health**						
Poor to fair	1184	14.2%	12.6%	90.3 (1), *p* <.001	7.6%	16.2%
Good to excellent	7148	85.8%	25.2%		92.4%	83.8%
**Self-rated Mental Health**						
Poor to fair	639	7.7%	7.2%	101.5 (1), *p* <.001	2.4%	9.3%
Good to excellent	7693	92.3%	24.8%		97.6%	90.7%
**Sex**						
Male	3995	47.9%	24.7%	6.6 (1), *p* <.02	50.5%	47.2%
Female	4337	52.1%	22.3%		49.5%	52.8%
**Age groups (years)**						
55-59	1197	14.4%	24.4%	23.0 (5), *p* <.001	15.0%	14.2%
60-64	2119	25.4%	24.4%		26.4%	25.1%
65-69	1738	20.9%	25.8%		23.0%	20.2%
70-74	1193	14.3%	23.6%		14.5%	14.3%
75-79	1263	15.2%	19.6%		12.7%	15.9%
80+	822	9.9%	20.1%		8.5%	10.3%
**Education**						
< Secondary school graduation	622	7.5%	16.7%	22.3 (2), *p* <.001	5.3%	8.1%
Secondary school graduate and/or with some post-secondary education	1613	19.4%	21.8%		18.0%	19.8%
Post-secondary degree/ Diploma	6097	73.2%	24.5%		76.7%	72.1%
**Mortgage**						
Paying rent	1769	21.2%	17.0%	62.6 (2), *p* <.001	15.4%	23.0%
Paying mortgage	1860	22.3%	22.5%		21.4%	22.6%
Paid off mortgage	4703	56.4%	26.2%		63.2%	54.4%
**Poverty-line status**						
Under poverty-line income	600	7.2%	10.2%	124.4 (3), *p* <.001	3.1%	8.4%
Marginal income	2408	28.9%	19.5%		24.1%	30.4%
Above poverty-line income	4719	56.6%	27.4%		66.4%	53.7%
No answer	605	7.3%	20.7%		6.4%	7.5%
**Marital status**						
Single	749	9.0%	16.8%	81.5 (3), *p* <.001	6.5%	9.8%
Married	5073	60.9%	26.6%		69.1%	58.4%
Widowed	1154	13.9%	21.4%		12.7%	14.2%
Divorced/ Separated	1356	16.3%	16.9%		11.7%	17.7%
**BMI**						
Underweight/ Normal weight	2252	27.0%	25.7%	19.1 (2), *p* < .001	29.7%	26.2%
Overweight	3296	39.6%	24.2%		40.8%	39.2%
Obese	2784	33.4%	20.7%		29.5%	34.6%
**Smoking status**						
Never smoked	2335	28.0%	25.0%	29.3 (2), *p* <.001	29.9%	27.5%
Former smoker	5313	63.8%	23.8%		64.8%	63.5%
Current smoker	684	8.2%	15.2%		5.3%	9.1%
**Sitting activity**						
Never/seldom	118	1.4%	23.7%	.007 (1), *p* = .936	1.4%	1.4%
Sometimes/often	8214	98.6%	23.4%		98.6%	98.6%
**Walking**						
Never/seldom	2733	32.8%	21.0%	13.2 (1), *p* < .001	29.4%	33.8%
Sometimes/often	5599	67.2%	24.6%		70.6%	66.2%
**Light/Moderate/Strenuous sports**						
No sports at all	6035	72.4%	22.0%	24.9 (1), *p* <.001	68.0%	73.8%
Played sports	2297	27.6%	27.2%		32.0%	26.2%
**Muscle & endurance exercises**						
Never/seldom	6815	81.8%	22.9%	4.5 (1), *p* < .05	80.2%	82.3%
Sometimes/often	1517	18.2%	25.5%		19.8%	17.7%
**Sleep problem**						
Never/rarely/some of the time	4903	58.8%	25.6%	32.2 (1), *p* <.001	64.4%	57.2%
Occasional/all of the time	3429	41.2%	20.3%		35.6%	42.8%
**Diabetes**						
No	6427	77.1%	25.1%	41.9 (1), *p* <.001	82.5%	75.5%
Yes	1905	22.9%	17.9%		17.5%	24.5%
**Heart disease**						
No	6958	83.5%	24.2%	15.6 (1), *p* <.001	86.4%	82.6%
Yes	1374	16.5%	19.3%		13.6%	17.4%
**Hypertension**						
No	4468	53.6%	25.5%	22.7 (1), *p* < .001	58.3%	52.2%
Yes	3864	46.4%	21.0%		41.7%	47.8%
**Arthritis**						
No	7274	87.3%	23.9%	6.1 (1), *p* < .02	88.9%	86.8%
Yes	1058	12.7%	20.4%		11.1%	13.2%
**Osteoporosis**						
No	7250	87.0%	24.2%	18.8 (1), *p* <.001	89.9%	86.1%
Yes	1082	13.0%	18.2%		10.1%	13.9%

#### Research Question 1: Which domains of adversity at baseline (e.g., physical, psychological and emotional, social, and self-rated wellness) are most conducive to regaining well-being?

Respondents who did not have physical wellness at baseline were less likely to achieve optimal well-being compared to those who had physical wellness at baseline (18.4% vs 24.8%; *χ2*(1) = 31.9; *p* < 0.001). A similar pattern was seen for psychological and emotional wellness (16.1% vs 33.2%; *χ2*(1) = 331.5; *p* < 0.001), social wellness (21.2% vs 26.1%; *χ2*(1) = 27.6; *p* < 0.001), and self-rated wellness (13.9% vs 26.2%; *χ2*(1) = 120.8; *p* < 0.001).

**Fig 2:**
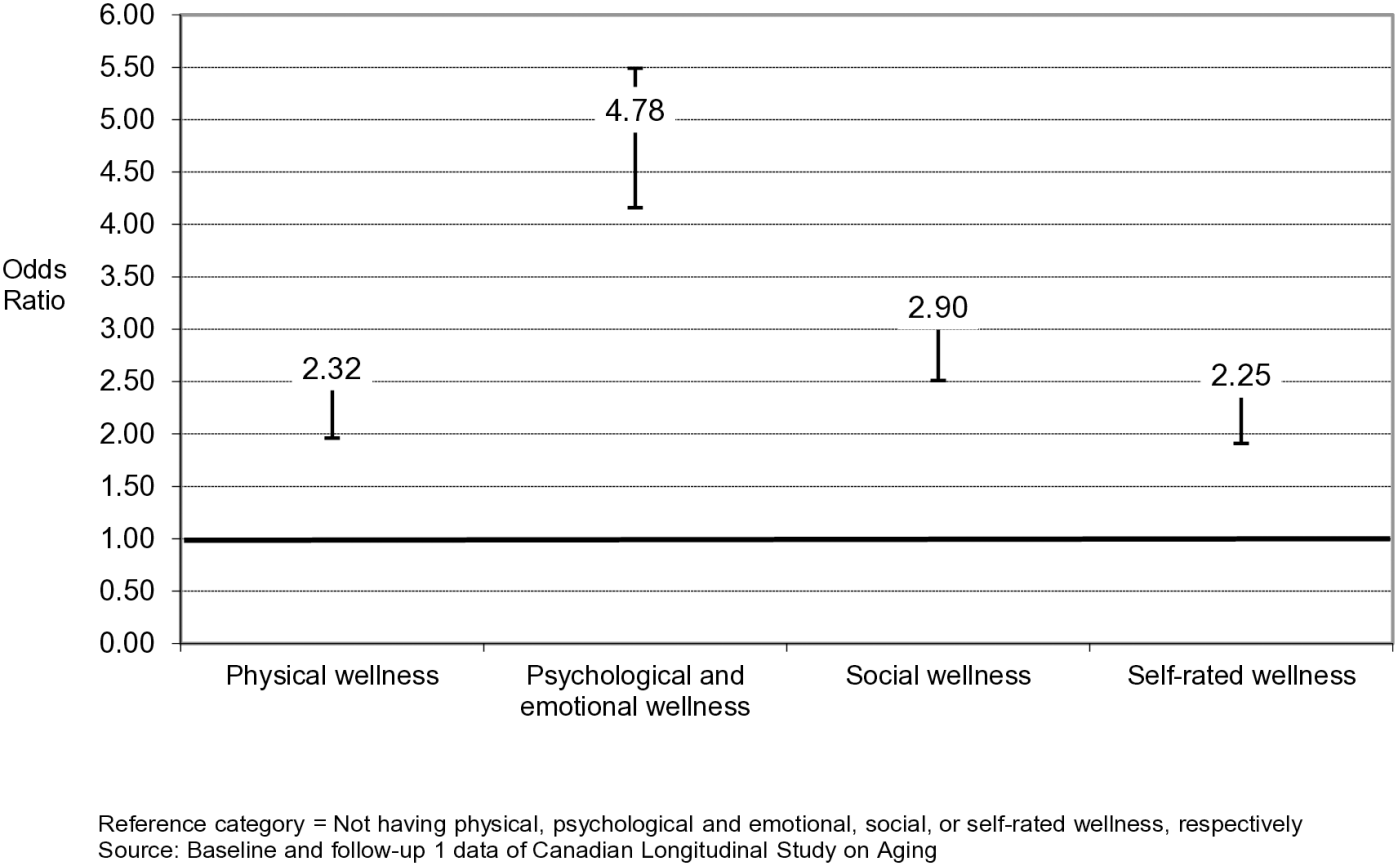
Fully adjusted odds ratio and 95% confidence interval of those not in optimal well-being at baseline and regaining optimal well-being at time 2 among respondents who were well in the wellness domains vs. respondents who were not well in these domains (*n* = 8332).

The results of the binary logistic regression models (Table 2 and Fig 2) confirmed that the age-sex adjusted odds of achieving optimal well-being at time 2 were higher among respondents who, at baseline, were in a state of physical wellness (Model 2: aOR = 2.42, 95% CI: 2.06, 2.85), psychological and emotional wellness (Model 2: aOR = 5.03, 95% CI: 4.39, 5.76), social wellness (Model 2: aOR = 3.21, 95% CI: 2.79, 3.69), and self-rated wellness (Model 2: aOR = 2.60, 95% CI: 2.22, 3.05) compared to those who were not well in these areas at baseline.

**Table 2:** Logistic regression analyses of baseline characteristics associated with achieving optimal well-being at time 2. Sample restricted to respondents not in optimal well-being at baseline (*n* = 8332). Adjusted odds ratios and 95% confidence intervals provided.

Variables	Model 1	Model 2	Model 3
	Wellness domains only	Wellness Domains + Age & Sex	Fully Adjusted
	*R*^*2*^ = 0.14	*R*^*2*^ = 0.15	*R*^*2*^ = 0.17
**Physical Wellness** (ref. no physical wellness)	**2.46 (2.09, 2.90)**	**2.42 (2.06, 2.85)**	**2.32 (1.96, 2.74)**
**Psychological and Emotional Wellness** (ref. no psychological and emotional wellness)	**4.89 (4.28, 5.59)**	**5.03 (4.39, 5.76)**	**4.78 (4.16, 5.49)**
**Social Wellness** (ref. no social wellness)	**3.29 (2.86, 3.78)**	**3.21 (2.79, 3.69)**	**2.90 (2.51, 3.34)**
**Self-rated Wellness** (ref. no self-rated wellness)	**2.55 (2.18, 3.00)**	**2.60 (2.22, 3.05)**	**2.25 (1.91, 2.65)**
**Female** (ref. male)		0.98 (0.88, 1.09)	1.07 (0.95, 1.20)
**Age groups** (ref. 80+)			
55-59		**1.50 (1.20, 1.89)**	**1.48 (1.16, 1.90)**
60-64		**1.43 (1.16, 1.77)**	**1.41 (1.12, 1.77)**
65-69		**1.49 (1.20, 1.87)**	**1.49 (1.18, 1.88)**
70-74		1.23 (0.97, 1.56)	1.24 (0.97, 1.58)
75-79		0.95 (0.74, 1.21)	0.96 (0.75, 1.23)
**Education** (ref. < secondary school graduation)			
Secondary school graduate and/or with some post-secondary education			1.08 (0.84, 1.40)
Post-secondary degree/ diploma			1.18 (0.94, 1.49)
**Wealth Measure** (ref. paying rent)			
Paying mortgage			0.93 (0.78, 1.12)
Paid off mortgage			1.05 (0.90, 1.23)
**Poverty-Line Status** (ref. under poverty-line income)			
Marginal income			**1.72 (1.26, 2.35)**
Above poverty-line income			**2.04 (1.49, 2.80)**
No answer			**1.77 (1.23, 2.55)**
**Marital status** (ref. single)			
Married			**1.46 (1.16, 1.84)**
Widowed			**1.44 (1.09, 1.90)**
Divorced/ Separated			1.13 (0.86, 1.47)
**BMI** (ref. obese)			
Underweight/ Normal weight			1.08 (0.93, 1.25)
Overweight			1.05 (0.92, 1.20)
**Smoking status** (ref. current smoker)			
Never smoked			**1.67 (1.31, 2.13)**
Former smoker			**1.57 (1.25, 1.98)**
**Sitting activities** (ref. never/seldom)			0.92 (0.59, 1.43)
**Walking** (ref. never/seldom)			1.03 (0.92, 1.16)
**Light/Moderate/Strenuous sports** (ref. no sports at all)			1.11 (0.98, 1.25)
**Muscle or endurance exercises** (ref. never/seldom)			1.09 (0.95, 1.25)
**Sleep problem** (ref. occasionally/all of the time)			1.01 (0.90, 1.13)
**Diabetes** (ref. with condition)			**1.25 (1.08, 1.45)**
**Heart disease** (ref. with the condition)			1.05 (0.90, 1.23)
**Hypertension** (ref. with the condition)			1.05 (0.94, 1.18)
**Arthritis** (ref. with the condition)			1.12 (0.94, 1.35)
**Osteoporosis** (ref. with the condition)			1.15 (0.96, 1.37)

Remarks: Numbers in bold indicate that *p*-value < .05

In the Full Model, which adjusted for 22 baseline factors (Fig 1 and Table 2 ), the adjusted odds of achieving optimal well-being at time 2 for respondents who, at baseline, had shown physical wellness (Model 3: aOR = 2.32, 95% CI: 1.96, 2.74), psychological and emotional wellness (Model 3: aOR = 4.78, 95% CI: 4.16, 5.49), social wellness (Model 3: aOR = 2.90, 95% CI: 2.51, 3.34) and self-rated wellness (Model 3: aOR = 2.25, 95% CI: 1.91, 2.65) were similar to the age-sex adjusted models, suggesting that the factors included in the fully adjusted model failed to attenuate the robust association between the four wellness domains at baseline, and optimal well-being at time 2. Additional analyses were conducted to investigate the association between the number of wellness domains achieved at baseline and regaining optimal well-being at time 2. It was found that participants who achieved at least two wellness domains were highly likely to regain optimal well-being at time 2 (1 wellness domain: not statistically significant; 2 wellness domains: aOR = 12.77, 95% CI: 3.25, 50.14; 3 wellness domains: aOR = 38.87, 95% CI: 9.94, 152.03). Results of the analysis are available in S3 Table in the Supporting Information.

The probability of achieving optimal well-being at time 2 was extremely low for those who had either ADL limitations and/or IADL limitations compared with those who were without either ADL or IADL limitations (i.e., 9.4% vs 24.0%; *χ2*(1) = 38.8; *p* < 0.001), and among those with depression as classified by the CES-D score [29–30] vs those without depression (i.e., 13.1% vs 27.1%; *χ2*(1) = 175.2; *p* < 0.001), and among those who were not happy at baseline compared to those who were happy (i.e., 14.8% vs 25.3%; *χ2*(1) = 75.5; *p* < 0.001). Similarly, among those who were not satisfied with life at baseline compared to those who were satisfied with life (i.e., 15.1% vs 25.9%; *χ2*(1) = 95.6; *p* < 0.001). Those who did not have social wellness at baseline were less likely to achieve optimal well-being compared to those who had social wellness (i.e., 21.2% vs 26.1%; *χ2*(1) = 27.6; *p* < 0.001), and those who rated their mental health as poor to fair were less likely to achieve optimal well-being compared to those who rated it as good to excellent (i.e., 7.2% vs 24.8%; *χ2*(1) = 101.5; *p* < 0.001). Those who, at baseline, rated their own aging as poor to fair and those who rated their physical health as poor to fair were less likely to achieve optimal well-being compared to those who rated these as good to excellent at baseline (i.e., own aging: 12.5% vs 25.0%; *χ2*(1) = 79.8; *p* < 0.001; physical health: 12.6% vs 25.2%; *χ2*(1) = 90.3; *p* < 0.001).

Individuals with lower socioeconomic characteristics were also less likely to achieve optimal well-being compared to those with higher socioeconomic characteristics. For example, those living under the poverty line had less than half the likelihood of achieving optimal well-being in comparison to those above the poverty line (i.e., 10.2% vs 27.4%; *χ2*(3) = 124.4; *p* < 0.001). Those without a high school diploma were less likely to achieve optimal well-being than those with a post-secondary degree/diploma (i.e., 16.7% vs 24.5%; *χ2*(2) = 22.3; *p* < 0.001). With respect to demographic characteristics, the prevalence of achieving optimal well-being was significantly lower among never-married respondents vs married respondents (i.e., 16.8% vs 26.6%; *χ2*(3) = 81.5; *p* < 0.001). Those who were 80 years old and older were less likely to achieve optimal well-being compared to those who were 55–64 years old (i.e., 20.1% vs 24.4%; *χ2*(5) = 23.0; *p* < 0.001).

Those who were obese at baseline were less likely to achieve optimal well-being compared to those who were underweight or had normal weight at baseline (i.e., 20.7% vs 25.7%; *χ2*(2) = 19.1; *p* < 0.001). Respondents who were smokers at baseline were less likely to achieve optimal well-being compared to those who never smoked (i.e., 15.2% vs 25.0%; *χ2*(2) = 29.3; *p* < 0.001). Those who never or seldom took a walk outside their home or yard for exercise or any reason at baseline were less likely to achieve optimal well-being compared to those who sometimes or often walked (i.e., 21.0% vs 24.6%; *χ2*(1) = 13.2; *p* < 0.001). Respondents who did not engage in light/moderate/strenuous sports were less likely to achieve optimal well-being compared to those who did (i.e., 22.0% vs 27.2%; *χ2*(1) = 24.9; *p* < 0.001). Similarly, those who did not perform muscle and endurance exercises were less likely to achieve optimal well-being (i.e., 22.9% vs 25.5%; *χ2*(1) = 4.5; *p* < 0.05). Respondents who had sleep problems were less likely to achieve optimal well-being compared to those who slept well (i.e., 20.3% vs 25.6%; *χ2*(1) = 32.2; *p* < 0.001). Those who had chronic diseases at baseline such as arthritis (i.e., 20.4% vs 23.9%; *χ2*(1) = 6.1; *p* < 0.02), diabetes (17.9% vs 25.1%; *χ2*(1) = 41.9; *p* < 0.001), heart disease (19.3% vs 24.2%; *χ2*(1) = 15.6; *p* < 0.001), hypertension (21.0% vs 25.5%; *χ2*(1) = 22.7; *p* < 0.001) and osteoporosis (18.2% vs 24.2%; *χ2*(1) = 18.8; *p* < 0.001) were less likely to achieve optimal well-being in comparison to those who did not have these diseases at baseline.

#### Research question 2: What factors/behaviors/characteristics at baseline are associated with regaining optimal well-being approximately three years after baseline?

In the fully adjusted model (see Table 2), other significant baseline factors associated with optimal well-being included being young (under 70 years old), having a higher income (above poverty-line income), being married or widowed, not currently smoking, and not having diabetes. Four separate analyses (see Tables 3 and 4) were conducted to examine factors associated with improved physical, psychological and emotional, social, and self-rated wellness.

**Table 3:** Adjusted odds ratios for improvement in four wellness domains at time 2 based on binary logistic regression.

Variables	Physical Wellness *n* = 1788	Psychological & Emotional Wellness *n* = 4775	Social Wellness *n* = 4499	Self-Rated Wellness *n* = 1867
	Fully Adjusted Model	Fully Adjusted Model	Fully Adjusted Model	Fully Adjusted Model
	*R*^*2*^ = 0.18	*R*^*2*^ = 0.07	*R*^*2*^ = 0.16	*R*^*2*^ = 0.20
**Physical Wellness** (ref. no physical wellness)	N/A	**1.70 (1.40, 2.06)**	1.01 (0.82, 1.24)	**1.40 (1.11, 1.78)**
**ADL/IADL Limitations** (ref. had ADL limitations only)				
No ADL nor IADL limitations	**3.46 (1.82, 6.60)**	N/A	N/A	N/A
No ADL limitations, but had IADL limitations	0.94 (0.53, 1.67)	N/A	N/A	N/A
**Disabling pain or discomfort** (ref. with disabling pain or discomfort)	**2.25 (1.27, 3.98)**	N/A	N/A	N/A
**Psychological and Emotional Wellness** (ref. no psychological and emotional wellness)	**1.39 (1.11, 1.74)**	N/A	**1.26 (1.09, 1.45)**	**2.17 (1.73, 2.73)**
**Depression** (ref. having depression as classified by the CES-D score^29–30^)	N/A	**0.83 (0.71, 0.96)**	N/A	N/A
**Felt depressed** (ref. felt depressed)	N/A	1.02 (0.86, 1.20)	N/A	N/A
**Felt happy** (ref. not felt happy)	N/A	1.00 (0.87, 1.15)	N/A	N/A
**Felt satisfied with life** (ref. not felt satisfied with life)	N/A	0.89 (0.77, 1.01)	N/A	N/A
**Social Wellness** (ref. no social wellness)	0.98 (0.79, 1.23)	**1.27 (1.11, 1.46)**	N/A	**1.51 (1.22, 1.86)**
**Had someone to give advice about a crisis** (ref. no one to give advice about a crisis)	N/A	N/A	**2.14 (1.86, 2.47)**	N/A
**Had someone to show love and affection** (ref. no one to show love and affection)	N/A	N/A	**2.67 (2.24, 3.18)**	N/A
**Had someone to confide or talk about a problem** (ref. no one to confide or talk about a problem)	N/A	N/A	**2.55 (2.22, 2.93)**	N/A
**Self-rated Wellness** (ref. no self-rated wellness)	**1.80 (1.41, 2.28)**	**1.89 (1.58, 2.25)**	1.07 (0.89, 1.30)	N/A
**Self-rated own aging** (ref. self-rated own aging not good)	N/A	N/A	N/A	**1.49 (1.21, 1.85)**
**Self-rated health** (ref. self-rated health not good)	N/A	N/A	N/A	**2.62 (2.08, 3.30)**
**Self-rated mental health** (ref. self-rated mental health not good)	N/A	N/A	N/A	**1.53 (1.20, 1.97)**
**Female** (ref. male)	0.89 (0.71, 1.12)	**0.85 (0.73, 0.98)**	**1.40 (1.21, 1.61)**	0.90 (0.72, 1.14)
**Age groups** (ref. 80+)				
55-59	1.19 (0.77, 1.85)	0.84 (0.62, 1.15)	**1.37 (1.03, 1.82)**	1.53 (0.93, 2.51)
60-64	1.08 (0.73, 1.59)	0.82 (0.62, 1.09)	1.28 (0.99, 1.64)	**1.63 (1.01, 2.62)**
65-69	1.23 (0.83, 1.84)	1.01 (0.76, 1.36)	1.19 (0.92, 1.54)	1.38 (0.85, 2.23)
70-74	0.77 (0.51, 1.18)	1.03 (0.76, 1.39)	1.13 (0.86, 1.50)	1.52 (0.91, 2.53)
75-79	0.96 (0.63, 1.46)	1.05 (0.77, 1.43)	0.84 (0.65, 1.10)	1.16 (0.70, 1.91)
**Education** (ref. < secondary school graduation)				
Secondary school graduate and/or with some post-secondary education	**1.77 (1.17, 2.67)**	0.92 (0.69, 1.21)	1.06 (0.81, 1.38)	1.23 (0.84, 1.81)
Post-secondary degree/ diploma	**1.64 (1.12, 2.39)**	0.92 (0.71, 1.19)	1.09 (0.85, 1.38)	1.12 (0.79, 1.58)
**Wealth Measure** (ref. paying rent)				
Paying mortgage	0.78 (0.57, 1.09)	1.06 (0.87, 1.29)	1.03 (0.84, 1.27)	1.19 (0.88, 1.60)
Paid off mortgage	1.20 (0.90, 1.60)	1.04 (0.87, 1.24)	0.95 (0.79, 1.13)	0.94 (0.72, 1.24)
**Poverty-Line Status** (ref. under poverty-line income)				
Marginal income	0.79 (0.52, 1.20)	**1.41 (1.05, 1.90)**	**1.42 (1.06, 1.90)**	0.86 (0.59, 1.26)
Above poverty-line income	0.64 (0.41, 1.00)	**1.57 (1.15, 2.14)**	**1.83 (1.35, 2.48)**	0.70 (0.46, 1.05)
No answer	0.67 (.039, 1.15)	**1.54 (1.06, 2.23)**	**1.70 (1.18, 2.43)**	0.82 (0.49, 1.37)
**Marital status** (ref. never-married)				
Married	**1.63 (1.05, 2.54)**	1.14 (0.88, 1.48)	**1.77 (1.38, 2.28)**	0.74 (0.50, 1.09)
Widowed	1.69 (0.99, 2.87)	**1.40 (1.02, 1.93)**	**1.40 (1.04, 1.89)**	0.96 (0.58, 1.57)
Divorced/ Separated	1.62 (0.99, 2.63)	1.06 (0.79, 1.42)	**1.32 (1.01, 1.74)**	**0.62 (0.41, 0.95)**
**BMI** (ref. obese)				
Underweight/ Normal weight	**1.42 (1.06, 1.90)**	0.86 (0.72, 1.03)	0.99 (0.83, 1.19)	1.31 (0.98, 1.75)
Overweight	**1.50 (1.18, 1.90)**	0.92 (0.79, 1.08)	0.95 (0.81, 1.11)	1.18 (0.93, 1.49)
**Smoking status** (ref. current smoker)				
Never smoked	**2.16 (1.42, 3.29)**	**1.43 (1.10, 1.87)**	1.05 (0.82, 1.35)	**1.48 (1.03, 2.13)**
Former smoker	**1.85 (1.24, 2.75)**	1.22 (0.95, 1.56)	1.11 (0.88, 1.40)	1.34 (0.97, 1.86)
**Sitting activities** (ref. never/seldom)	0.53 (0.23, 1.26)	0.67 (0.41, 1.09)	0.77 (0.47, 1.29)	0.98 (0.43, 2.26)
**Walking** (ref. never/seldom)	1.15 (0.93, 1.41)	1.07 (0.93, 1.23)	1.05 (0.92, 1.21)	**1.48 (1.20, 1.83)**
**Light/Moderate/Strenuous sports** (ref. no sports at all)	0.82 (0.64, 1.05)	**1.16 (1.00, 1.34)**	1.01 (0.87, 1.17)	0.99 (0.77, 1.27)
**Muscle or endurance exercises** (ref. never/seldom)	0.92 (0.71, 1.19)	1.05 (0.89, 1.25)	**1.23 (1.04, 1.45)**	**1.51 (1.12, 2.03)**
**Sleep problem** (ref. occasionally/all of the time)	**1.32 (1.07, 1.62)**	0.94 (0.82, 1.08)	1.02 (0.89, 1.17)	**1.53 (1.25, 1.87)**
**Diabetes** (ref. with condition)	1.14 (0.90, 1.45)	**1.19 (1.01, 1.40)**	1.03 (0.88, 1.22)	1.17 (0.93, 1.46)
**Heart disease** (ref. with the condition)	0.83 (0.63, 1.10)	1.15 (0.95, 1.40)	0.88 (0.74, 1.06)	1.24 (0.96, 1.59)
**Hypertension** (ref. with the condition)	1.23 (1.00, 1.52)	1.01 (0.88, 1.15)	1.05 (0.92, 1.21)	0.81 (0.65, 1.00)
**Arthritis** (ref. with the condition)	**1.49 (1.13, 1.95)**	1.09 (0.89, 1.34)	1.12 (0.90, 1.40)	1.22 (0.92, 1.63)
**Osteoporosis** (ref. with the condition)	**1.64 (1.21, 2.22)**	1.01 (0.84, 1.23)	**1.36 (1.11, 1.67)**	1.01 (0.75, 1.38)

Remarks: Numbers in bold indicate that *p*-value < .05

**Table 4:** Baseline factors associated with improvement in four wellness domains.

	Physical Wellness Improvement	Psychological and Emotional Wellness Improvement	Social Wellness Improvement	Self-Rated Wellness Improvement
Had physical wellness at baseline	N/A	✓		✓
Had psychological and emotional wellness at baseline	✓	N/A	✓	✓
Had social wellness at baseline		✓	N/A	✓
Had self-rated wellness at baseline	✓	✓		N/A
Being young			✓	
Being female			✓	
Having higher education	✓	✓	✓	
Being married	✓		✓	
Not being obese	✓			
Not smoking	✓	✓		✓
Being physically active		✓	✓	✓
Not having sleeping problems	✓			✓
Living without chronic disease(s)	✓	✓	✓	

In the analysis restricted to the 1788 respondents who did not have physical wellness at baseline, the results of the binary logistic regression models predicting the restoration of physical wellness at time 2 confirmed that the fully adjusted odds of achieving physical wellness at time 2 were higher among respondents who, at baseline, had no ADL nor IADL limitations (aOR = 3.46, 95% CI: 1.82, 6.60), had no disabling pain or discomfort (aOR = 2.25, 95% CI: 1.27, 3.98), had psychological and emotional wellness (aOR = 1.39, 95% CI: 1.11, 1.74), and had self-rated wellness (aOR = 1.80, 95% CI: 1.41, 2.28) compared to those who were not well in these areas at baseline. Other significant baseline factors associated with improved physical wellness included having higher education (more than secondary school education), being married, not being obese, not currently smoking, not having sleeping problems, and living without arthritis and osteoporosis.

In the psychological and emotional wellness-specific analysis, restricted to the 4775 respondents who did not have psychological and emotional wellness at baseline, the fully adjusted odds of achieving psychological and emotional wellness at time 2 were higher among respondents who had physical wellness (aOR = 1.70, 95% CI: 1.40, 2.06), had social wellness (aOR = 1.27, 95% CI: 1.11, 1.46) and had self-rated wellness (aOR = 1.89, 95% CI: 1.58, 2.25), while having depression as classified by the CES-D score [29–30] was negatively associated with restoring psychological and emotional wellness by time 2 (aOR = 0.83, 95% CI: 0.71, 0.96). Other significant baseline factors associated with reclaiming psychological and emotional wellness by time 2, included having a higher income (above poverty-line income), being widowed, never smoking, engaging in light, moderate or strenuous sports, and not having diabetes.

In the social wellness-specific analysis, of the 4499 respondents who did not have social wellness at baseline, the fully adjusted odds of restoring social wellness at time 2 were higher among respondents who had psychological wellness (aOR = 1.26, 95% CI: 1.09, 1.45), had someone to give advice about a crisis (aOR = 2.14, 95% CI: 1.86, 2.47), had someone to show love and affection (aOR = 2.67, 95% CI: 2.24, 3.18), and had someone to confide or talk about a problem (aOR = 2.55, 95% CI: 2.22, 2.93). Other significant baseline factors associated with reclaiming social wellness at time 2 included being young (under 60 years old), female, having a higher income (above poverty-line income), not never-married, engaging in muscle or endurance exercises, and not having osteoporosis.

In the self-rated wellness-specific analysis, of the 1867 respondents who did not have self-rated wellness at baseline, the fully adjusted odds of achieving self-rated wellness at time 2 were higher among respondents who, at baseline, had physical wellness (aOR = 1.40, 95% CI: 1.11, 1.78), had psychological and emotional wellness (aOR = 2.17, 95% CI: 1.73, 2.73), had social wellness (aOR = 1.51, 95% CI: 1.22, 1.86), had good self-rated own aging (aOR = 1.49, 95% CI: 1.21, 1.85), had good self-rated health (aOR = 2.62, 95% CI: 2.08, 3.30), and had good self-rated mental health (aOR = 1.53, 95% CI: 1.20, 1.97) while being separated or divorced was negatively associated with improvement in self-rated wellness (aOR = 0.62, 95% CI: 0.41, 0.95). Other baseline factors associated with improved self-rated wellness included never smoking, walking, engaging in muscle or endurance exercises and not having sleeping problems.

### Assessment of model fit

The results of the Omnibus Tests of Model Coefficients were highly significant (all respondents’ model: *χ2*(34) = 988.1; *p* < .001; physical wellness’ model: *χ2*(36) = 255.1; *p* < .001; psychological and emotional wellness’ model: *χ2*(37) = 233.7; *p* < .001; social wellness’ model: *χ2*(36) = 551.8; *p* < .001; and self-rated wellness’ model: *χ2*(36) = 305.2; *p* < .001), indicating that the final model in each analysis is significantly better than the unadjusted baseline model. The Nagelkerke’s *R*^2^ values ranged from 0.07 to 0.20 in the models (all respondents’ model: Nagelkerke’s *R*^2^ = 0.17; physical wellness’ model: Nagelkerke’s *R*^2^ = 0.18; psychological and emotional wellness’ model: Nagelkerke’s *R*^2^ = 0.07; social wellness’ model: Nagelkerke’s *R*^2^ = 0.16; and self-rated wellness’ model: Nagelkerke’s *R*^2^ = .20), implying that the final model explains between 6.5% to 20.1% of the variation in respective outcome. All variance inflation factors (VIFs) of the predictor variables in all five models (i.e., all respondents’ model, physical wellness’ model, psychological and emotional wellness’ model, social wellness’ model, and self-rated wellness’ model) ranged from 1.01 to 2.12 (VIF < 10), indicating that multicollinearity was not a concern.

## Discussion

Guided by the integrative conceptual framework composed of the ecological systems theory, [21,22] Keyes’ concept of complete mental health [23], and Young *et al.*’s multidimensional model of successful aging, [24] the definition of optimal well-being used in this study is comprised of four wellness domains: (1) physical wellness – not having limitations in ADLs and IADLs, disabling chronic pain or discomfort, (2) psychological and emotional wellness – the absence of any serious mental illness, memory problems or low mood, (3) social wellness – the presence of adequate social support, and (4) self-rated wellness – older adults’ subjective perception of their aging process, physical health, mental health and emotional well-being, regardless of the number of chronic diseases present [13–15]

This study examined the prevalence and characteristics of respondents who did not meet the criteria for optimal well-being at baseline, yet improved and ultimately achieved optimal well-being by time 2. Nearly one-quarter of these individuals regained optimal well-being across the three-year study period. Of particular interest, this study identified which domains of adversity (i.e., physical, psychological and emotional, social, and self-rated wellness) were most conducive to regaining optimal well-being over the duration of the study. The findings indicated that respondents with baseline psychological and emotional wellness were the most likely to regain optimal well-being. They were almost five times more likely to achieve optimal well-being at time 2 than those who did not have psychological and emotional wellness at baseline. Other significant baseline factors included being younger (under 70 years old), [35] having a higher income (above poverty-line income), [36] not smoking, [37] and living without diabetes [38]. These relationships remained almost the same after adjustment for 22 additional factors.

These findings were consistent with recent studies on healthy aging [39,40], which noted the importance of psychological and social well-being in achieving optimal well-being in later life. The disease-oriented medical system often focuses on diagnosing and treating specific diseases, while neglecting the broader context of older adults’ health and well-being, particularly in the domains of psychological well-being (e.g., happiness and life satisfaction), [41] and social well-being (e.g., maintaining healthy relationships and social connections) [42]. In keeping with findings of our study, other research has also indicated that healthy aging encompasses more than just physical health status, but also psychological, social, and self-rated well-being [43,44]. Additional studies have suggested that healthy aging is more common among those with greater purpose in life, [39] religiosity and spirituality [45,46], and among those with playful personalities [47,48]. Future research is needed to further investigate these factors and their impact on achieving optimal well-being in later life.

This study also examined baseline factors associated with improvement in four wellness domains. The wellness domains were positively associated with each other. For example, respondents who had wellness in any of the other three wellness domains at baseline were more likely to achieve psychological and emotional wellness or self-rated wellness at time 2. For the physical wellness domain, respondents who, at baseline, had no ADL nor IADL limitations, disabling chronic pain or discomfort, were more likely to recover physically. Carpentieri and colleagues found that older adults used different strategies to maximize their well-being as they aged [49]. For the psychological and emotional wellness domain, respondents who had depression as classified by the CES-D score [29–30] at baseline were less likely to recover psychologically and emotionally. For the social wellness domain, respondents with someone to show love and affection at baseline were more likely to recover socially. Previous studies also showed the importance of fostering psychological and social wellness in promoting optimal aging [50–51]. For the self-rated wellness domain, respondents with good self-rated health at baseline were almost three times as likely to recover in the self-rated wellness domain than those who had rated their health less than good at baseline. Subjective well-being, particularly life satisfaction (or evaluative well-being), feelings of happiness (hedonic well-being), and meaning in life or sense of purpose (eudemonic well-being), were found to be a determinant of optimal health as people age [52]. It was important to note that a few significant baseline factors associated with improvement were modifiable lifestyle factors such as not being obese, not smoking, engaging in physical activity, and not having sleeping problems, as found in previous studies [13–15] In addition, successful prevention of chronic disease(s) (e.g., arthritis, diabetes, and osteoporosis) may also help individuals reclaim optimal health.

Previous longitudinal research suggests older adults who, at baseline, had greater physical resilience, [53] psychosocial resources, [54] and/or subjective well-being, [55] including having hope [56], were more likely to demonstrate resilience and regain optimal well-being. The findings of our current study provide further evidence that aging is neither a linear path nor synonymous with deterioration. Older adults can rebound from challenges and struggles to reclaim optimal health. If future research establishes that the associations observed in the current study are causal, there is a need for effective policies and interventions that support physical, psychological and emotional, social, and self-rated wellness. These interventions may play an important role in enhancing older adults' resilience and enabling them to regain optimal well-being in later life.

### Limitations

The following limitations should be considered when interpreting the findings of this study. Firstly, the study took place in Canada, where a universal, publicly funded healthcare system is in place [57]. Under the Canada Health Act, all Canadian citizens and permanent residents have reasonable access to medically necessary services at no cost. The study’s findings may not fully apply to other countries, particularly those where patients must pay for healthcare services. Future research is needed in other high-income countries, such as the USA, which do not offer lifelong free medical care. It is also unclear whether the findings can be generalized to low- and middle-income countries. Secondly, as discussed elsewhere, [13–15] the construction of the optimal well-being status variable used in this secondary data analysis study was restricted by variables available in the CLSA data. For example, subjective well-being could have been studied further if variables on eudemonic well-being (i.e., meaning in life or sense of purpose) were available. Thirdly, respondents in the CLSA were more educated than the general population, with 79.5% of the CLSA sample holding a post-secondary degree compared to less than half of Canadians aged 65 years and over (45%) [58]. Despite these limitations, the analyses of baseline and time 2 of the CLSA data provided valuable information on how people change over time and what baseline factors are associated with the regaining of optimal well-being in later life.

## Conclusions

The findings of this study are encouraging as they show that one-quarter of older adults with less than optimal well-being can improve to the point where they regain optimal well-being. Our identification of factors at baseline which are associated with regaining optimal well-being suggests the potential value of pursuing strategies related to engaging in an active and healthy lifestyle, such as maintaining healthy body weight, not smoking, becoming physically active, finding ways to tackle sleeping problems, preventing and managing chronic diseases, as well as maintaining psychological and emotional wellness, and fostering social wellness. Most of these are modifiable lifestyle factors that can be achieved without geographic limitations. Furthermore, programs and services can be provided to support older adults to engage in an active and healthy lifestyle (e.g., nutrition and fitness classes, smoke free campaigns); manage chronic conditions (e.g., chronic disease self-management programs, cognitive behavioral theory for insomnia programs); prevent physical disabilities (e.g., fall prevention programs, road safety awareness); promote mental health (e.g., counselling programs, support groups); and prevent social isolation (e.g., congregate dining programs, friendly visiting services). Policies and funding focusing on these areas may support older adults in achieving optimal well-being in later life. Future research should be conducted to understand the needs of older adults and to evaluate the impact of such programs and services.

Our findings underline the possibility of improvements in physical and mental well-being, even in later life. When we work together, older adults can thrive in a society that genuinely values aging and focuses on older adults’ strengths rather than their limitations.

## Supporting information

S1 Table Description of covariates at baseline. (PDF)

S2 Table Description of outcomes. (PDF)

S3 Table Adjusted odds ratios for number of wellness domain at baseline based on binary logistic regression (*n* = 8332). (PDF)
