# Supplementary material for: Reclaiming wellness: Key factors in restoring optimal well-being in the Canadian Longitudinal Study on Aging
Source: PLoS One. 2025 Sep 24;20(9):e0329800. doi: 10.1371/journal.pone.0329800 (PMC12459853; doi:10.1371/journal.pone.0329800)
Supplement: S1 Table — (PDF) [file pone.0329800.s001.pdf]

S1 Table. Description of covariates at baseline[1]

Variable	Definition
Age groups	Based on a question about the respondent's age in years, the response was divided into six categories: 55-59, 60-64, 65-69, 70-74, 75-79, 80+.
Sex	Based on a question about the respondent's biological sex, the response was divided into two categories: male, female.
Marital status	Based on a question about the respondent's current marital/partner status, the response was divided into five categories: single, never married or never lived with a partner; married or living with a partner, common-law relationship; widowed; divorced or separated.
Education factors	Based on a question about the respondent's highest level of education, the response was divided into three categories: less than secondary school graduation; secondary school graduate and/or some post-secondary education; post-secondary degree/diploma.
Wealth measure	Based on a question about the respondent's ownership of the principal residence, the response was divided into three categories: paying rent, paying the mortgage, and paying off the mortgage.
Poverty line status	Calculated by comparing the respondent's household income and household size with the poverty line in 2015 when the data collection of time 2 began,[2] the response was divided into three categories: under poverty line income, marginal income, above poverty line income.
BMI	Based on a question about the respondent's BMI, the response was divided into three categories: underweight (BMI < 18.49999) or normal weight (BMI >=18.5 but < 24.99999), overweight (BMI >= 25 but < 29.99999), obese (BMI >= 30).
Smoking status	Based on a question about the respondent's type of smoker, the response was divided into three categories: never smoked, former smoker, current smoker.
Sitting activities	Based on the question, "Over the past 7 days, how often did you participate in sitting activities such as reading, watching TV, computer activities or doing handicrafts?" the response was divided into two categories: never or seldom, sometimes or often.
Walking	Based on the question, "Over the past 7 days, how often did you take a walk outside your home or yard for any reason? For example, for pleasure or exercise, walking to work, walking the dog, etc." the response was divided into two categories: never or seldom, sometimes or often.
Light/Moderate/Strenuous sports	Based on the three questions on light sports – "Over the past 7 days, how often did you engage in light sports or recreational

	activities such as bowling, golf with a cart, shuffleboard, badminton, fishing or other similar activities?" moderate sports – "Over the past 7 days, how often did you engage in moderate sports or recreational activities such as ballroom dancing, hunting, skating, golf without a cart, softball or other similar activities?" and strenuous sports – "Over the past 7 days, how often did you engage in strenuous sports or recreational activities such as jogging, swimming, snowshoeing, cycling, aerobics, skiing, or other similar activities?" respectively the responses were divided into two categories: no sports at all, played sports.
Muscle and endurance exercises	Based on the question, "Over the past 7 days, how often did you do any exercises specifically to increase muscle strength and endurance, such as lifting weights or push-ups, etc.?" the response was divided into two categories: never or seldom, sometimes or often.
Sleep problem	Based on the question, "How often was your sleep restless?" the response was divided into two categories: never or rarely, some of the time, occasionally or all of the time.
Diabetes	Based on a yes/no question that asked if the respondent had ever been told by a doctor that s/he had diabetes, borderline diabetes or that your blood sugar was high. Coded as "no" if answered "no" to this question.
Heart disease	Based on a yes/no question that asked if the respondent had ever been told by a doctor that s/he had heart disease (including congestive heart failure, or CHF). Coded as "no" if answered "no" to this question.
Hypertension	Based on a yes/no question that asked if the respondent had ever been told by a doctor that s/he had high blood pressure or hypertension. Coded as "no" if answered "no" to this question.
Arthritis	Based on a yes/no question that asked if the respondent had ever been told by a doctor that s/he had any other type of arthritis. Coded as "no" if answered "no" to this question.
Osteoporosis	Based on a yes/no question that asked if the respondent had ever been told by a doctor that s/he had osteoporosis, sometimes called low bone mineral density, or thin, brittle or weak bones. Coded as "no" if answered "no" to this question.

Reference

1. Ho M, Pullenayegum E, Burnes D, Fuller-Thomson E. Successful Aging among Immigrant and Canadian-Born Older Adults: Findings from the Canadian Longitudinal Study on Aging (CLSA). *International Journal of Environmental Research and Public Health*. 2022;19(20): 13199. https://doi.org/10.3390/ijerph192013199

2. Statistics Canada. Low-Income measure (LIM) thresholds by income source and household size. 2015. Accessed 28 August, 2022. https://www150.statcan.gc.ca/t1/tbl1/en/tv.action?pid=1110023201
