# Supplementary material for: Reclaiming wellness: Key factors in restoring optimal well-being in the Canadian Longitudinal Study on Aging
Source: PLoS One. 2025 Sep 24;20(9):e0329800. doi: 10.1371/journal.pone.0329800 (PMC12459853; doi:10.1371/journal.pone.0329800)
Supplement: S2 Table — (PDF) [file pone.0329800.s002.pdf]

S2 Table. Description of outcomes[1]

Variable	Definition
Limitations in Activity of Daily Living (ADL)	Based on yes/no response to five questions on (1) ability to dress and undress oneself without help; (2) ability to eat without help; (3) ability to walk without help; (4) ability to walk with some help from a person or with the use of a walker or crutches, etc.; (5) ability to get in and out of bed without any help or aids. Coded as "no" if answered "no" to all of the five questions.
Limitations in Instrumental Activity of Daily Living (IADL)	Based on yes/no response to eight questions on (1) ability to use the telephone without help; (2) ability to get to places out of walking distance without help; (3) ability to go shopping for groceries or clothes without help; (4) ability to prepare own meals without help; (5) ability to do housework without help; (6) ability to do housework with some help; (7) ability to take own medicine without help; (8) ability to handle own money without help. Coded as "no" if answered "no" to all of the eight questions.
Disabling pain or discomfort	Derived from responses to two questions that asked if respondents were usually free of pain or discomfort (yes/no) and the number of activities prevented by the pain or discomfort (none, a few, some, most). Coded as "no" if "free from pain or discomfort" and "none or a few activities prevented by the pain or discomfort."
Mental disorders – Anxiety, Depression, Posttraumatic Stress Disorder (PTSD)	Derived from responses to three questions that were (1) based on yes/no response to a question that asked if respondents had ever been told by a doctor that they had an anxiety disorder such as a phobia, obsessive-compulsive disorder or a panic disorder; (2) based on the Centre for Epidemiological Studies Short Depression Scale (CES-D 10) score, coded as not having depression if the CES-D 10 score < 10;[2-3] (3) coded as not having PTSD based on the Primary Care Posttraumatic Stress Disorder (PC-PTSD) score < 3.[4]
Low mood – Felt depressed, felt happy, felt satisfied with life	Derived from responses to three questions that asked how often respondents felt depressed; felt happy; and felt satisfied with life (all of the time (5-7 days), occasionally (3-5 days), some of the time (1-2 days), rarely or never (less than 1 day)). Coded as "no" if the respondents answered, "felt depressed rarely or never, or some of the time", "felt happy occasionally or all of the time", and "felt satisfied with life occasionally or all of the time."
Lack of social support	Derived from responses to three questions that asked if respondents had (1) someone to give advice about a crisis; (2) someone who showed love and affection; (3) someone to confide in or talk to about oneself or one's problems (none of the time, a little of the time, some of the time, most of the time, all of the

	time).[33] Coded as "no" if the respondents answered "most of the time or all of the time" in all three questions.
Lack of self-rated wellness	Derived from responses to three questions that asked respondents to rate their (1) aging process; (2) perception of physical health; and (3) perception of mental health. Coded as "no" if the respondents answered "good to excellent" in all three questions.
Physical wellness	Derived from responses to yes/no questions on (1) Limitations in ADL; (2) Limitations in IADL; and (3) Disabling pain or discomfort. Coded as "yes" if the respondents answered "no" to all of these questions.
Psychological and emotional wellness	Derived from responses to yes/no question on (1) Mental disorders; and (2) Low mood. Coded as "yes" if the respondents answered "no" to all of these questions.
Social wellness	Derived from responses to yes/no question on (1) Lack of social wellness. Coded as "yes" if the respondents answered "no" to this question.
Self-rated wellness	Derived from responses to yes/no question on (1) Lack of self-rated wellness. Coded as "yes" if the respondents answered "no" to this question.
Optimal well-being	Derived from responses to yes/no question on (1) Physical wellness; (2) Psychological and emotional wellness; (3) Social wellness; and (4) Self-rated wellness. Coded as "yes" if the respondents answered "yes" to all of these questions.

Reference

1. Ho M, Pullenayegum E, Burnes D, Fuller-Thomson E. Successful Aging among Immigrant and Canadian-Born Older Adults: Findings from the Canadian Longitudinal Study on Aging (CLSA). *International Journal of Environmental Research and Public Health*. 2022;19(20): 13199. https://doi.org/10.3390/ijerph192013199

2. Hann D, Winter K, Jacobsen P. Measurement of depressive symptoms in cancer patients. Evaluation of the Center for Epidemiological Studies Depression Scale (CES-D). *Journal of Psychosomatic Research*. 1999;46:437-443. https://doi.org/10.1016/s0022-3999(99)00004-5

3. Radloff LS. The CED-D scale: A self-report depression scale for research in the general population. *Applied Psychological Measurement*. 1977;1:385-401. https://doi.org/10.1177/014662167700100306

4. Prins A, Bovin MJ, Smolenski DJ, et al. The Primary Care PTSD Screen for DSM-5 (PC-PTSD-5): Development and evaluation within a veteran primary care sample. *Journal of General Internal Medicine*. 2016;31:1206-1211. https://doi.org/10.1007/s11606-016-3703-5
