# Supplementary material for: Reclaiming wellness: Key factors in restoring optimal well-being in the Canadian Longitudinal Study on Aging
Source: PLoS One. 2025 Sep 24;20(9):e0329800. doi: 10.1371/journal.pone.0329800 (PMC12459853; doi:10.1371/journal.pone.0329800)
Supplement: S3 Table — (PDF) [file pone.0329800.s003.pdf]

S3 Table. Adjusted odd ratios for number of wellness domain at baseline based on binary logistic regression (*n* = 8332)

Variables	Model 1	Model 2	Model 3
	Number of Wellness domains only	Number of Wellness Domains + Age & Sex	Fully Adjusted
	$R^2 = 0.12$	$R^2 = 0.13$	$R^2 = 0.15$
**Number of Wellness Domains** (ref. 0 wellness domain at baseline)			
1 wellness domain	**4.36 (1.07, 17.75)**	**4.29 (1.05, 17.48)**	4.06 (1.00, 16.57)
2 wellness domains	**15.44 (3.94, 60.48)**	**15.37 (3.92, 60.24)**	**12.77 (3.25, 50.14)**
3 wellness domains	**50.31 (12.90, 196.24)**	**50.28 (12.89, 196.18)**	**38.87 (9.94, 152.03)**
**Female** (ref. male)		0.91 (0.82, 1.02)	0.99 (0.89, 1.12)
**Age groups** (ref. 80+)			
55-59		**1.37 (1.09, 1.72)**	**1.38 (1.08, 1.76)**
60-64		**1.30 (1.05, 1.60)**	**1.28 (1.02, 1.61)**
65-69		**1.39 (1.12, 1.73)**	**1.39 (1.11, 1.75)**
70-74		1.15 (0.91, 1.46)	1.16 (0.91, 1.47)
75-79		0.92 (0.72, 1.18)	0.93 (0.73, 1.19)
**Education** (ref. < secondary school graduation)			
Secondary school graduate and/or with some post-secondary education			1.04 (0.81, 1.34)
Post-secondary degree/ diploma			1.14 (0.91, 1.44)
**Wealth Measure** (ref. paying rent)			
Paying mortgage			0.92 (0.77, 1.10)
Paid off mortgage			1.08 (0.93, 1.27)
**Poverty-Line Status** (ref. under poverty-line income)			
Marginal income			**1.70 (1.25, 2.33)**
Above poverty-line income			**1.98 (1.44, 2.71)**
No answer			**1.77 (1.23, 2.54)**
**Marital status** (ref. single)			
Married			**1.42 (1.14, 1.78)**
Widowed			**1.39 (1.06, 1.84)**
Divorced/ Separated			1.08 (0.83, 1.41)
**BMI** (ref. obese)			
Underweight/ Normal weight			1.01 (0.87, 1.17)
Overweight			1.00 (0.87, 1.13)
**Smoking status** (ref. current smoker)			
Never smoked			**1.65 (1.30, 2.10)**

Former smoker			**1.53 (1.21, 1.92)**
**Sitting activities**<br>(ref. never/seldom)			0.95 (0.61, 1.48)
**Walking**<br>(ref. never/seldom)			1.02 (0.91, 1.15)
**Light/Moderate/Strenuous sports**<br>(ref. no sports at all)			1.11 (0.98, 1.25)
**Muscle or endurance exercises**<br>(ref. never/seldom)			1.09 (0.95, 1.25)
**Sleep problem**<br>(ref. occasionally/all of the time)			1.06 (0.95, 1.19)
**Diabetes**<br>(ref. with condition)			**1.24 (1.08, 1.43)**
**Heart disease**<br>(ref. with the condition)			1.03 (0.88, 1.20)
**Hypertension**<br>(ref. with the condition)			1.04 (0.93, 1.16)
**Arthritis**<br>(ref. with the condition)			1.08 (0.90, 1.29)
**Osteoporosis**<br>(ref. with the condition)			1.16 (0.98, 1.39)
